# Individual *HLA-A, -B, -C*, and *-DRB1* Genotypes Are No Major Factors Which Determine COVID-19 Severity

**DOI:** 10.3389/fimmu.2021.698193

**Published:** 2021-07-26

**Authors:** Johannes Schetelig, Falk Heidenreich, Henning Baldauf, Sarah Trost, Bose Falk, Christian Hoßbach, Ruben Real, Axel Roers, Dirk Lindemann, Alexander Dalpke, Martin Kolditz, Katja de With, Martin Bornhäuser, Ezio E. Bonifacio, Elke Rücker-Braun, Vinzenz Lange, Jan Markert, Ralf Barth, Jan A. Hofmann, Jürgen Sauter, Stefanie N. Bernas, Alexander H. Schmidt

**Affiliations:** ^1^ Clinical Trials Unit, DKMS, Dresden, Germany; ^2^ Division of Hematology, Department of Internal Medicine I, University Hospital Carl Gustav Carus, Technische Universität (TU), Dresden, Dresden, Germany; ^3^ Institute for Immunology, TU Dresden, Dresden, Germany; ^4^ Institute of Medical Microbiology and Virology, University Hospital Carl Gustav Carus, TU Dresden, Dresden, Germany; ^5^ Division of Pulmonology, Department of Internal Medicine I, University Hospital Carl Gustav Carus, TU Dresden, Dresden, Germany; ^6^ Division of Infectious Diseases, TU Dresden, Dresden, Germany; ^7^ Center for Regenerative Therapies Dresden (CRTD), TU Dresden, Dresden, Germany; ^8^ DKMS Life Science Lab, Dresden, Germany; ^9^ DKMS, Stem Cell Donor Registry, Tübingen, Germany

**Keywords:** HLA, SARS-CoV-2, immunogenetics, *in silico* prediction, T-cell epitopes

## Abstract

HLA molecules are key restrictive elements to present intracellular antigens at the crossroads of an effective T-cell response against SARS-CoV-2. To determine the impact of the *HLA* genotype on the severity of SARS-CoV-2 courses, we investigated data from 6,919 infected individuals. HLA-A, -B, and -DRB1 allotypes grouped into HLA supertypes by functional or predicted structural similarities of the peptide-binding grooves did not predict COVID-19 severity. Further, we did not observe a heterozygote advantage or a benefit from HLA diplotypes with more divergent physicochemical peptide-binding properties. Finally, numbers of *in silico* predicted viral T-cell epitopes did not correlate with the severity of SARS-CoV-2 infections. These findings suggest that the *HLA* genotype is no major factor determining COVID-19 severity. Moreover, our data suggest that the spike glycoprotein alone may allow for abundant T-cell epitopes to mount robust T-cell responses not limited by the *HLA* genotype.

## Introduction

T-cell recognition is central for the adaptive immune response to a new challenge such as SARS-CoV-2. Once a virus has invaded a cell and becomes integrated in the cell’s protein synthesis machinery, processing of translated virus proteins allows for presentation of the foreign intracellular antigens to CD8^+^ T cells *via* HLA class I molecules by most nucleated cells. In addition, endocytosed viral proteins are presented to CD4^+^ T cells on HLA class II complexes of professional antigen-presenting cells which also cross-present endocytosed antigen on HLA class I molecules to CD8^+^ T cells. Presentation of viral peptides on HLA molecules is an essential step required for adaptive immunity to the virus and therefore critically determines the clinical course (1–4). The antigen processing machinery cleaves viral proteins into peptides and determines the output of peptides for loading onto HLA complexes. Each HLA allotype confers the ability to bind and present a distinct spectrum of peptides. For SARS-CoV-2 infections many findings support the critical role of a robust initial T-cell response (1, 3, 5). Circulating SARS-CoV-2-specific CD8^+^ and CD4^+^ T cells have been identified experimentally by several independent groups in convalescent patients (3, 6, 7). Multiple SARS-CoV-2 derived HLA class I and class II presented peptides have been identified and characterized as potential T-cell epitopes (3, 6, 7). CD4^+^ and CD8^+^ T-cell epitopes map to all major viral proteins suggesting a robust and diverse T-cell response (6, 7). The breadth and magnitude of SARS-CoV-2-specific HLA-DR T-cell responses correlated with antibody titers and greater diversity of SARS-CoV-2 T-cell responses was associated with less severe courses (3). Notably, certain SARS-CoV-2 peptides elicit memory T-cell responses even in unexposed individuals (3). These data provide a hint to cross-reactive T-cell immunity between SARS-CoV-2 and ‘common cold’ coronaviruses, including human coronavirus (HCoV)-OC43, HCoV-229E, HCoV-NL63, and HCoV-HKU1 (3, 8, 9).

The individual potential to present virus epitopes is restricted by the HLA genotype. The composition of the set of *HLA* alleles is highly individualized raising the hypothesis that the genotype itself could determine the T-cell response to a given virus. This has been demonstrated for chronic infections such as hepatitis B, hepatitis C, and human immunodeficiency virus (HIV) (10–13). No consistent data are available on the impact of specific *HLA* genotypes on the course of acute viral infections (14–16). Investigating this question for SARS-CoV-2 seems promising against the backdrop of highly different clinical courses of this acute infection.

DKMS is a stem cell donor registry that administrates *HLA* data from volunteers for hematopoietic stem cell donation relevant for donor-patient matching (17). In response to the pandemic, we launched a population-based study to identify risk factors for severe COVID-19 courses. The specific aim of this study was to determine for particular HLA allotypes if they boost or impede T-cell mediated immune responses against SARS-CoV-2.

## Methods

### Study Design

The project was designed as a registry-based cross-sectional study. Existing immunogenetic data were linked to self-reported COVID-19-specific data collected with a standardized health questionnaire. The responsible Institutional Review Board of the Technische Universität Dresden (IRB00001473) approved the study. We registered the study with the trial registry of the German Center for Infection Research (https://dzif.clinicalsite.org/de/cat/2099/trial/4361). Data privacy of the participating individuals was protected in accordance with the General Data Protection Regulation of the European Union. We conducted the study in compliance with the principles of the Declaration of Helsinki. All participants provided explicit consent that COVID-19-specific data were linked to immunogenetic data in the DKMS donor registry file.

### Phenotype Definitions for COVID-19 Severity

Symptomatic infections were defined by any symptom which occurred together with the diagnosis of the SARS-CoV-2 infection. Severe respiratory symptoms were defined by the combination of at least fever and cough, dyspnea and cough, dyspnea and fever, or dyspnea and myalgia. Respiratory hospitalizations were defined by in-patient care with supplemental oxygen or mechanical ventilation or hospitalization for dyspnea or cough.

### HLA Genotyping

At the time of registration to the DKMS registry, volunteers provided buccal swabs or blood for DNA extraction and genotyping. The standard genotyping of a panel of genes relevant for stem cell donor selection including information on *HLA-A, -B, -C, -DRB1* was predominantly performed by the DKMS Life Science Lab applying a high-resolution amplicon-based approach using Illumina devices (18, 19).

### HLA Supertypes

*HLA-A* and *HLA-B* alleles were clustered into supertypes based on their ability to bind specific amino acid residues of peptides in the binding groove, as defined for the two main pockets, B and F (20). *HLA-DRB1* alleles were assigned to supertypes based on common structural and functional features of HLA class II molecules to define the classes DR1, DR3, DR4, DR5, and DR9 according to Doytchinova and Flower (21).

### Homozygosity and Physicochemical Divergence of HLA Diplotypes

Homozygosity at the *HLA-A, -B, -C*, and/or *-DR* locus was determined at the two-field level. To test for an impact of the diversity of the combined immunopeptidomes of two given *HLA*-class I alleles we calculated the Grantham Distance as a surrogate measure for the evolutionary divergence of *HLA-A, -B*, and -*C* diplotypes (22–24). The Grantham Distance is a measure to compare physicochemical properties (composition, polarity, and molecular volume) of amino acid sequences. The more divergent two HLA alleles are in their peptide-binding pouch, reflected by a larger Grantham Distance, the broader the repertoire of peptides that might be presented to immune effector cells. The current understanding is that the observed HLA allelic diversity was partly driven by an evolutionary advantage of more divergent alleles.

### Viral Proteins

The 14 protein reference sequences of the SARS-CoV-2 virus (strain Wuhan-Hu-1) were downloaded from the UniProt database (proteome UP000464024). This set comprises the structural proteins spike glycoprotein (P0DTC2), membrane protein (P0DTC5), nucleoprotein (P0DTC9), envelope small membrane protein (P0DTC4) and the 10 accessory proteins 3a (P0DTC3), replicase polyprotein 1ab (P0DTD1), replicase polyprotein 1a (P0DTC1), ORF6 protein (P0DTC6), ORF7a protein (P0DTC7), ORF7b protein (P0DTD8), ORF8 protein (P0DTC8), ORF9b protein (P0DTD2), ORF10 protein (A0A663DJA2), and uncharacterized protein 14 (P0DTD3).

### Peptide Retrieval

For the retrieval of peptide sequences that are likely to be presented by HLA molecules, we used the integrated functions of NetMHCpan v4.1 (25) and NetMHCIIpan v4.0 (26) to generate all possible peptides of a pre-defined length of 8 to 12 amino acids (AA) for HLA class I and 13 to 24 AAs for HLA class II. Out of this pool consisting of 77,285 HLA class I peptides and 183,954 HLA class II peptides, we excluded duplicates (28,230 HLA class I and 67,446 HLA class II peptides) and 434 HLA class I and 1,776 HLA class II peptides spanning 7 mutation hotspots with an entropy of >0.25 retrieved from the Global Initiative on Sharing All Influenza Data database (GISAID, https://www.gisaid.org/): ORF1a(b) T265I; ORF1a(b) G392D; ORF1a(b) A876T; ORF1ab P4714L; spike glycoprotein D614G; ORF3a Q57H; and membrane protein T175M (4, 27). Further, we excluded 703 HLA class I and 2,987 HLA class II peptides which are not likely to be generated since they span one of the 14 NSP cleavage sites (https://www.uniprot.org). Finally, we excluded 33 HLA class I peptides that mimic peptides from human proteins since these are not expected to elicit immune responses (28). Of the remaining peptides we defined four pools: all peptides (ALL), peptides originating from highly expressed and stabilized proteins (N, M, S, NSP1, NSP5, NSP8, ORF9b) (29) (HIGHEX), peptides derived from the spike glycoprotein only (SPIKE), and peptides generated from conserved regions (30) (CONS).

The SPIKE pool was defined and analyzed in order to predict immunogenicity of the full-length spike-coding mRNA vaccines BNT162b2 by BioNTech/Pfizer and mRNA1273 by Moderna/NIAID. The viral strains circulating in Europe during the study period had a glycine (G) at position 614 instead of an aspartate (D). The D614-version of the spike glycoprotein is characteristic for the Wuhan-1-Hu strain and is coded by the two mRNA vaccines (31, 32). We excluded all peptides spanning at least one of the AA positions 614, 986 and 987. The latter two positions are exchanged by prolines in the vaccine to stabilize the spike glycoprotein in its pre-fusion conformation.

### Prediction of Strongly Binding Peptide-MHC Complexes

We used NetMHCpan v. 4.1 (25) and NetMHCIIpan v. 4.0 (26) for the prediction of peptide binding to HLA class I and class II molecules, respectively. These tools predict the binding affinity for a given peptide to an *HLA* allele, and rank it in a reference set. The percentile ranks are then used to define binding and non-binding peptides for each allele. The default system thresholds and scores were applied. We defined all HLA-assigned peptides with a rank score ≤0.5% as strong binders, and all others as non-binders. Exploratory analyses were performed with a rank score of 2%. For each *HLA* allele, the number of strong binding peptides was counted. The numbers of predicted strong binding peptides were then aggregated as peptide-MHC (pMHC) scores defined by the sum of the number of predicted pMHC complexes for the sets of *HLA-A, -B, -C* and *-DRB1* alleles for each individual. Identical peptides presented by different HLA-alleles or overlapping peptides presented by same HLA-alleles were counted separately because they would probably be recognized by different T-cell receptors providing additional chances to activate the immune system.

Some *HLA* alleles shared identical nucleotide sequences for peptide binding domains (coded by exons 2 and 3 for *HLA* class I and exon 2 only for *HLA* class II). Those alleles were grouped together, with the resulting group highlighted by a “G” attached to the third field of the lowest numbered allele in this group. We cross-checked the group assignment using neural networks prediction of binding affinity for SARS-CoV-2 peptides. With a test set of 736 SARS-CoV-2 peptides we predicted peptide presentation for the alleles of these “G” groups. Since all “G” alleles revealed the same binding, we reduced the “G” group to the first given second-field level of the allele group.

### Statistical Analysis and Power Calculations

To describe the distribution of data, median, range, and interquartile range (IQR) were used for continuous variables while frequencies and percentages were used for categorical variables. The predicted pMHC score and the Grantham Distance were analyzed categorically and continuously.

Binary logistic regression models were used to investigate different classification approaches with the risk of symptomatic infection, risk of severe respiratory symptoms and risk of respiratory hospitalization. Odds ratios (OR) and their 95% confidence intervals were used to describe the associations. Statistical testing was based on two-sided Wald tests for regression coefficients. All models were adjusted for sex, both age and age squared, both BMI and BMI squared, diabetes mellitus medication (yes/no), arterial hypertension medication (yes/no), and smoking status. In addition, tests for the risk of symptomatic infections were adjusted for the month of the positive test (January to July 2020). No interactions between main variables and covariates were identified at a significance level of 5%. Individuals with *HLA* data lacking a sufficient level of resolution for the respective classification approaches were excluded from analysis.

We evaluated the following biological concepts: heterozygote advantage, HLA evolutionary divergence, HLA supertypes, single allotypes, and *in silico* predicted peptide-MHC scores. The significance level was adjusted for the number of tests performed within the framework of a biological concept to maintain a false discovery rate of 5%. The number of tests to adjust for was determined by multiplying the number of *HLA* loci with the number of classifications, e.g. the number of supertypes, *HLA* alleles, or peptide pools. Adjusted p-values were calculated with the Benjamini-Hochberg procedure. Severe respiratory symptoms were tested as primary endpoint. Tests for symptomatic infections and respiratory hospitalizations were performed as explanatory analyses.

To detect odds ratios ≤0.5 or ≥2.0 for the risk of developing severe respiratory symptoms with a family-wise significance level of 5% this study had approximately 100% power for all 14 supertypes with a frequency of at least 10%, 92% for A01A24 (frequency of 5%), 72% for A01A03 (3%) and 43% for DR9 (2%). For the same effect sizes and significance level the power of testing homozygous vs. heterozygous allele pairs was 99% for *HLA-B* and 100% for *HLA-A, -C* and *-DRB1*. The power of testing presence vs. absence of the most common *HLA* alleles was between 82% at an allele frequency of 4%, e.g. HLA-A*31:01 and 100% at allele frequencies of at least 9%. All analyses were carried out using R Statistical Software version 3.5.1. Figures were created with R Statistical Software version 3.5.1 and BioRender.com.

## Results

### Medical Data

In a survey among registered stem cell donors conducted in August 2020, 157,544 participants reported results from SARS-CoV-2 tests including 7,948 participants who reported infections. We analysed the severity of infections in 6,919 individuals who reported positive tests between January and July 2020 (**Supplementary Figure S1**). Patient characteristics are shown in **Table 1**. Three nested phenotypes were constructed. Altogether 6,218 individuals (89.9%) reported symptomatic infections. This group included 1,821 individuals (26.3%) who reported symptoms indicating a severe respiratory tract infection of whom 266 patients (3.8%) needed hospitalization due to respiratory symptoms. Altogether, 161 participants needed supplemental oxygen and 22 participants needed mechanical ventilation. We assumed that viral susceptibility is not modulated by HLA molecules since the first steps of infection with a new virus (i.e. attachment, fusion, uncoating, and primary translation) are independent from HLA. Therefore, we decided not to analyze the risk of contracting SARS-CoV-2 by HLA genotype. Details on the data collection, demographic information of SARS-CoV-2 positive and negative participants and results of the analysis of clinical risk factors and ABO blood groups have been published previously (33, 34).

**Table 1:** Characteristics of SARS-CoV-2 positive participants.

Characteristic	N	(%)
**Total numbers**	6,919	(100)
**Sex**		
Female	4,637	(67.0)
Male	2,282	(33.0)
**Age in years**		
18-24	1,124	(16.2)
25-29	1,142	(16.5)
30-34	1,092	(15.8)
35-39	907	(13.1)
50-54	724	(10.5)
40-44	716	(10.3)
45-49	682	(9.9)
55-61	532	(7.7)
**Body Mass Index in kg/m²**		
<18.5	104	(1.5)
18.5 to < 25	3,791	(54.8)
25 to <30	1,963	(28.4)
30 to <35	749	(10.8)
35 to <40	222	(3.2)
≥40	90	(1.3)
**Diabetes mellitus**		
No	6,785	(98.1)
Yes	134	(1.9)
**Arterial hypertension**		
No	6,345	(91.7)
Yes	574	(8.3)
**Smoking status**		
Never	4,362	(63.0)
Past, not in 2019	1,560	(22.5)
Active in 2019	997	(14.4)
**Month of test**		
January/February	27	(0.4)
March	3,075	(44.4)
April	2,147	(31.0)
May	771	(11.1)
June	484	(7.0)
July	415	(6.0)
COVID-19 phenotype*		
Symptomatic infection	6,218	(89.9)
Severe respiratory symptoms	1,821	(26.3)
Respiratory hospitalization	266	(3.8)

* The three phenotypes are nested. Patients with respiratory hospitalization are a subset of participants with severe respiratory symptoms and participants with severe respiratory symptoms represent a subset of participants with symptomatic infection.

### HLA Supertypes Were Not Associated With COVID-19 Severity

**Figure 1:**
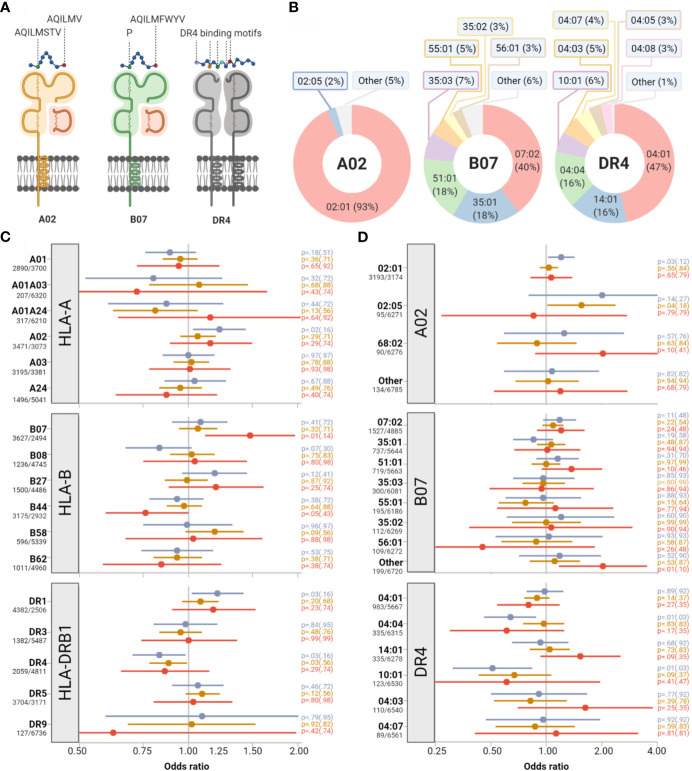
Impact of selected HLA supertypes and allotypes on COVID-19 severity. **(A)** Assignment of HLA allotypes into HLA-supertypes based on peptide binding characteristics. Supertype-specific amino acids at peptide anchor positions 2 and 9 are indicated with capital letters exemplary for supertypes A02 and B07. **(B)** The relative proportions of HLA alleles constituting supertypes A02, B07, and DR4 are depicted as doughnut charts. **(C, D)** Impact of supertypes and single alleles on symptomatic infections (blue), severe respiratory symptoms (orange) and respiratory hospitalizations (red). Point estimates for odds ratios (represented as colored dots) and corresponding 95% confidence intervals (represented by colored bars) illustrate the effects of presence versus absence of supertypes **(C)** and allotypes **(D)**. Absolute numbers of patients with presence/absence of the supertype/allotype are provided on the left side. p-values of Wald tests and in brackets adjusted p values for multiple testing are given on the right side.

Building on the hypothesis that the repertoire of immunogenic peptides derived from SARS-CoV-2 could be polarized toward particular steric and biochemical properties, we tested the impact of HLA supertypes on clinical outcome (20). HLA supertypes represent groups of allotypes, which share peptide binding specificity, as determined by defined molecular B-pocket and F-pocket structures for HLA-A and B (**Figures 1A, B**). None of the supertypes had a statistically significant impact on the primary outcome after stringent adjustment for multiple testing (**Figure 1C**). We detected weak signals toward more respiratory hospitalizations among individuals with a B07 supertype (OR 1.48, 95%-CI 1.11-1.97; p=0.01, p_adj_=0.14) and more symptomatic infections among individuals with the A02 supertype (OR 1.22, 95%-CI 1.03-1.44, p=0.02, p_adj_=0.16) and the DR1 supertype (OR 1.20, 95%-CI 1.02-1.42, p=0.03, p_adj_=0.16). Individuals with the supertype DR4 showed a weak trend to a lower risk of symptomatic infections (OR 0.83, 95%-CI 0.70-0.98; p=0.03, p_adj_=0.16).

### No Single HLA Allotype Showed a Strong Impact on COVID-19 Severity

Next, we dissected the allelic composition of the B07 and A02 supertypes (**Figures 1B, D**) and investigated the impact of the presence or absence of single allotypes on COVID-19 severity. Among allotypes constituting the A02 supertype, individuals with HLA-A*02:01 showed a trend toward greater risk of symptomatic infections (p=0.03, p_adj_=0.12) and those with HLA-A*02:05 toward a greater risk of severe respiratory infections (p=0.04, p_adj_=0.18). For HLA-A*02:05 the estimated effect directions, however, differed for the three nested clinical endpoints. Also, estimated effects for major allotypes which belonged to one HLA supertype did not homogeneously point in one direction (**Figure 1D**). A comparable set of partly incongruent results was found for allotypes constituting the B07, DR1, and the DR4 supertypes.

Further, we systematically investigated the impact of the 10 most common allotypes of *HLA-A, -B, -C*, and *-DRB1*. No single allotype showed even a trend for the risk of developing severe symptoms of a respiratory tract infection. The strongest association with a lower risk of symptomatic infections was found for HLA-C*07:01 (OR 0.73, 95%-CI 0.61-0.88, p=0.001, p_adj_=0.01). For the risk of respiratory hospitalization the strongest impact was seen for HLA-B*44:02 (OR 0.53, 95%-CI 0.34-0.85, p=0.008, p_adj_=0.08). Data on the impact of the 10 most common allotypes are shown in **Table 2**. Data on allotypes with population frequencies of more than 0.5% are provided in **Supplementary Table S1**.

**Table 2:** Impact of common HLA-A, -B, -C, -DRB1 alleles on COVID-19 severity.

Allele	N	(%)	Symptomatic infection				Severe respiratory symptoms				Respiratory hospitalization			
			OR	(95%-CI)	p	p_adj_	OR	(95%-CI)	p	p_adj_	OR	(95%-CI)	p	p_adj_
**HLA-A alleles**														
02:01	3,193	(50.1)	1.20	(1.02-1.42)	.029	.29	1.03	(0.92-1.16)	.56	.93	1.06	(0.82-1.38)	.65	.95
03:01	1,741	(27.3)	1.04	(0.86-1.25)	.70	.90	1.01	(0.89-1.15)	.86	.93	1.10	(0.82-1.47)	.54	.95
01:01	1,735	(27.2)	0.92	(0.76-1.10)	.35	.88	1.01	(0.89-1.15)	.84	.93	1.06	(0.79-1.42)	.72	.95
24:02	1,156	(18.2)	1.03	(0.82-1.27)	.82	.90	0.98	(0.85-1.14)	.81	.93	0.80	(0.55-1.15)	.22	.95
11:01	680	(10.7)	0.81	(0.63-1.04)	.10	.37	1.06	(0.88-1.27)	.54	.93	0.96	(0.62-1.48)	.84	.95
68:01	435	(6.8)	1.07	(0.76-1.50)	.69	.90	0.93	(0.74-1.17)	.54	.93	0.98	(0.58-1.66)	.95	.95
32:01	432	(6.8)	0.89	(0.65-1.22)	.47	.90	0.90	(0.72-1.13)	.38	.93	0.98	(0.58-1.66)	.94	.95
26:01	369	(5.8)	0.95	(0.68-1.33)	.76	.90	0.90	(0.70-1.15)	.40	.93	1.20	(0.71-2.04)	.49	.95
25:01	300	(4.7)	0.97	(0.66-1.44)	.90	.90	0.79	(0.60-1.04)	.095	.93	0.50	(0.22-1.14)	.099	.95
31:01	279	(4.4)	0.74	(0.51-1.07)	.11	.37	0.99	(0.75-1.30)	.93	.93	0.74	(0.36-1.52)	.41	.95
**HLA-B alleles**														
07:02	1,527	(23.9)	1.18	(0.96-1.45)	.11	.36	1.09	(0.95-1.24)	.22	.89	1.20	(0.89-1.61)	.24	.59
08:01	1,172	(18.4)	0.84	(0.68-1.03)	.098	.36	1.04	(0.90-1.21)	.55	.89	1.03	(0.74-1.45)	.84	.94
44:02	944	(14.8)	0.96	(0.76-1.21)	.72	.80	0.99	(0.84-1.16)	.88	.97	0.53	(0.34-0.85)	.008	.080
15:01	795	(12.5)	1.05	(0.82-1.35)	.70	.80	0.96	(0.80-1.14)	.62	.89	0.92	(0.61-1.39)	.68	.86
35:01	737	(11.5)	0.85	(0.66-1.09)	.19	.48	1.06	(0.89-1.27)	.48	.89	1.01	(0.67-1.53)	.94	.94
51:01	719	(11.3)	1.15	(0.88-1.50)	.31	.56	1.00	(0.83-1.19)	.97	.97	1.37	(0.94-2.01)	.10	.51
18:01	625	(9.8)	1.08	(0.81-1.44)	.59	.80	0.86	(0.71-1.05)	.14	.89	1.21	(0.79-1.84)	.38	.74
40:01	618	(9.7)	0.80	(0.61-1.04)	.094	.36	1.09	(0.91-1.32)	.35	.89	1.29	(0.85-1.95)	.23	.59
44:03	494	(7.7)	1.17	(0.85-1.62)	.34	.56	0.92	(0.74-1.14)	.44	.89	0.81	(0.48-1.38)	.44	.74
27:05	445	(7.0)	1.01	(0.73-1.39)	.97	.97	1.02	(0.82-1.28)	.83	.97	1.15	(0.70-1.89)	.58	.83
**HLA-C alleles**														
07:01	1,581	(26.0)	0.73	(0.61-0.88)	.001	.010	0.95	(0.83-1.08)	.44	.75	1.00	(0.74-1.37)	.98	.98
07:02	1,500	(24.7)	1.20	(0.98-1.47)	.085	.24	1.09	(0.95-1.24)	.22	.75	1.24	(0.91-1.67)	.17	.82
04:01	1,422	(23.4)	0.99	(0.81-1.21)	.91	.91	1.11	(0.97-1.27)	.13	.75	1.04	(0.76-1.43)	.80	.89
06:02	1,158	(19.0)	1.14	(0.91-1.42)	.27	.44	0.96	(0.82-1.11)	.56	.75	0.86	(0.60-1.23)	.42	.82
03:04	920	(15.1)	0.90	(0.71-1.13)	.34	.49	1.08	(0.92-1.26)	.36	.75	1.10	(0.76-1.59)	.61	.87
05:01	823	(13.5)	1.25	(0.96-1.63)	.096	.24	1.06	(0.90-1.25)	.49	.75	0.84	(0.55-1.27)	.40	.82
12:03	694	(11.4)	1.32	(0.99-1.76)	.057	.24	0.95	(0.79-1.14)	.60	.75	1.06	(0.70-1.60)	.79	.89
02:02	697	(11.5)	1.04	(0.80-1.36)	.76	.85	0.97	(0.81-1.17)	.77	.86	0.86	(0.55-1.33)	.49	.82
03:03	628	(10.3)	0.89	(0.68-1.16)	.39	.49	0.92	(0.76-1.12)	.40	.75	0.76	(0.47-1.23)	.26	.82
01:02	371	(6.1)	1.27	(0.87-1.87)	.21	.42	0.99	(0.77-1.26)	.91	.91	1.37	(0.83-2.26)	.22	.82
**HLA-DRB1 alleles**														
15:01	1,711	(25.7)	1.10	(0.91-1.34)	.32	.89	1.13	(1.00-1.28)	.048	.26	1.25	(0.95-1.66)	.11	.62
07:01	1,558	(23.4)	1.22	(1.00-1.49)	.056	.56	0.92	(0.80-1.05)	.20	.41	0.84	(0.61-1.15)	.28	.71
03:01	1,318	(19.8)	0.98	(0.80-1.21)	.85	.89	0.95	(0.82-1.09)	.46	.51	1.01	(0.73-1.38)	.97	.99
01:01	1,164	(17.5)	0.98	(0.79-1.21)	.84	.89	1.08	(0.94-1.25)	.27	.44	1.03	(0.74-1.44)	.87	.99
11:01	1,003	(15.1)	1.06	(0.84-1.34)	.61	.89	0.97	(0.83-1.13)	.70	.70	0.73	(0.49-1.09)	.12	.62
13:01	999	(15.0)	1.11	(0.88-1.40)	.38	.89	1.16	(1.00-1.35)	.051	.26	1.11	(0.78-1.58)	.55	.99
04:01	983	(14.8)	0.98	(0.78-1.24)	.89	.89	0.89	(0.76-1.04)	.14	.39	0.80	(0.54-1.18)	.27	.71
13:02	510	(7.7)	1.04	(0.76-1.42)	.81	.89	1.16	(0.94-1.42)	.16	.39	1.01	(0.63-1.63)	.95	.99
11:04	428	(6.4)	1.10	(0.78-1.56)	.57	.89	0.90	(0.72-1.14)	.38	.48	1.12	(0.67-1.86)	.66	.99
08:01	383	(5.8)	0.85	(0.61-1.20)	.36	.89	1.13	(0.90-1.43)	.30	.44	1.00	(0.57-1.74)	.99	.99

HLA, Human Leukozyte Antigen; N, number; OR, odds ratio; CI, confidence interval; p, p-value; padj, adjusted p-value.

### Heterozygosity and HLA Divergence Showed No Consistent Impact on Disease Severity

Homozygosity for *HLA* alleles reduces the spectrum of the viral peptides that can be presented and thus may limit the efficiency of adaptive immunity (11, 35). In a set of exploratory analyses we tested whether individuals homozygous for one or more HLA alleles were at greater risk of symptomatic SARS-CoV-2 infections and severe courses of COVID-19 compared to heterozygous individuals. Data on *HLA-A, -B*, and *-C* and *HLA-DRB1* at the two-field level were available for 5,773 and 6,650 individuals, respectively. In total 1,320 participants (22.9%) were homozygous for at least one *HLA* class I allele and 524 (7.9%) were homozygous for *HLA-DRB1*. Homozygosity for *HLA-A* was associated with a weak trend for greater risk of having severe respiratory symptoms (OR 1.18, 95%-CI 1.01-1.37; p=0.04, p_adj_=0.16). Homozygosity for *HLA-B, HLA-C* or *HLA-DRB1* did not show significant associations with more severe COVID-19 courses. While the number of homozygous loci did not show a monotonous correlation, individuals with three homozygous *HLA* loci had a significantly greater risk (OR 1.74, p=0.005, p_adj_=0.02) of showing severe respiratory symptoms from SARS-CoV-2 (see **Supplementary Table S2**). Fourteen individuals in this group (14/115, 12.2%) shared the HLA-A*03:01~HLA-B*07:02~HLA-C*07:02 haplotype and seven (50%) of those reported severe respiratory symptoms pointing toward the possibility of an effect linked to this specific haplotype rather than resulting from homozygosity.

**Figure 2:**
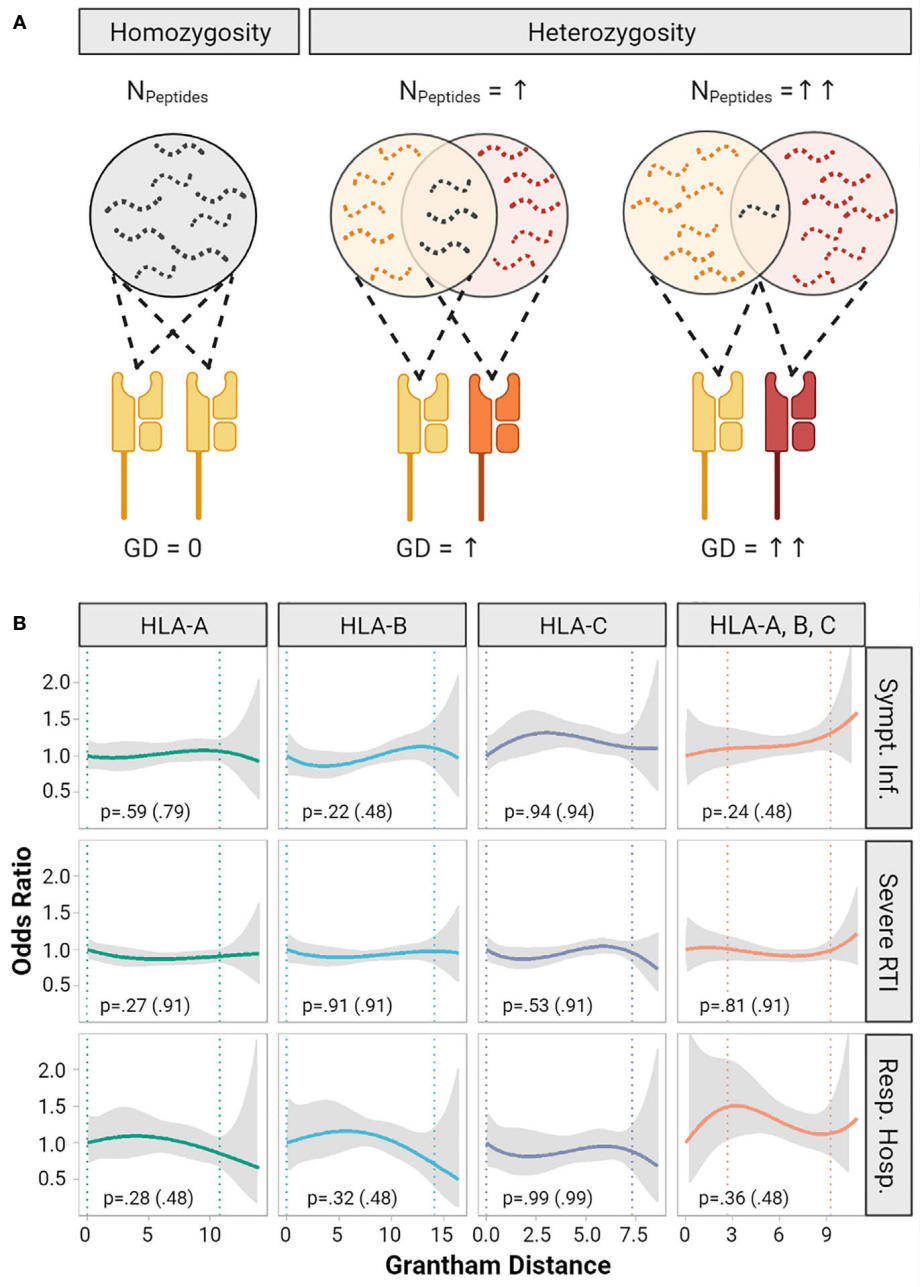
Impact of Grantham Distance of heterozygous HLA-A, - B, -C alleles on COVID-19 severity. **(A)** The concept of the evolutionary divergence of HLA (HED): For a given HLA-locus the pool of presentable peptides is limited in size and diversity, when both alleles are identical (i.e. homozygous). It gets larger for heterozygous allele combinations and might be largest for two HLA-alleles that show the highest HED. Divergence might be quantified by the Grantham Distance (GD), a metric based on physicochemical differences of the amino acid sequence of HLA-molecules. Panel **(B)** shows smoothed plots for the odds ratios (OR) of the risk of symptomatic infection, risk of severe respiratory symptoms (RTI) and risk of respiratory hospitalization depending on the GD of HLA-A, -B, -C and the mean of these three loci. The gray ribbons represent the pointwise 95% confidence intervals. Homozygous individuals with a GD of 0 were set as reference (OR = 1). The vertical dotted lines indicate a 90% range of individual’s GD by cutting the lowest and highest 5% of GD values observed. The p-values are based on multivariable logistic regression models testing whether a one-unit increase of the Grantham Distance increases the odds ratio. In addition, p values adjusted for multiple testing are provided in brackets.

The functional diversity of a given HLA diplotype to present diverse immunopeptidomes is defined by the allelic sequence divergence of the peptide-binding domains (**Figure 2A**). The Grantham Distance (GD) is a measure which allows quantification of physicochemical differences between protein amino acid sequences, here of HLA molecules constituting the diplotype (22). Recently, it was demonstrated that greater GD of HLA class I diplotypes correlated with improved survival after immune checkpoint inhibitor treatment for cancer, suggesting that T-cell mediated anti-tumor immunity partly depends on diversity of the HLA immunopeptidomes (23). In this cohort the median GD was 7.1 (IQR 4.5 to 10.5, range 0 to 14.0) for HLA-A, 8.4 (IQR 6.8 to 9.9, range 0 to 16.4) for HLA-B and 4.8 (IQR 3.4 to 6.5, range 0 to 8.6) for HLA-C (**Supplementary Figure S2**). We did not detect significant correlations of the GD with the risk of symptomatic infections, severe respiratory infections, or respiratory hospitalizations (**Figure 2B**).

### Severity of COVID-19 Courses Does Not Correlate With Number of *In Silico* Predicted High-Affinity Viral Peptide-MHC Complexes

**Figure 3:**
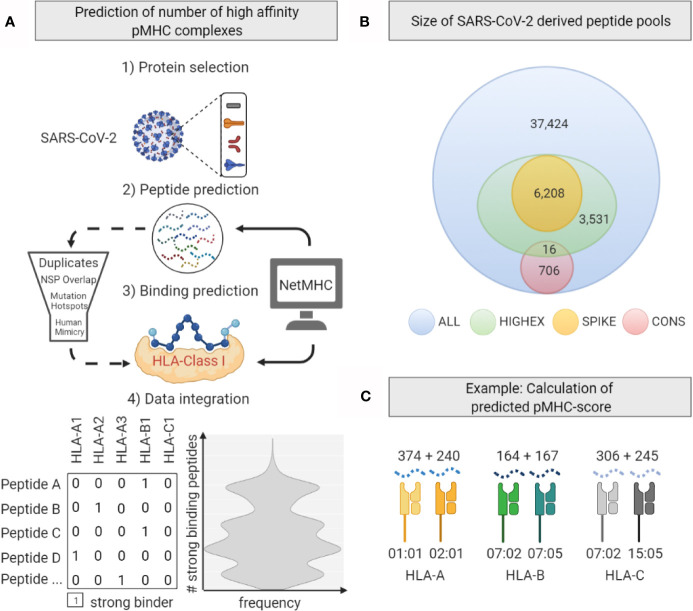
Generation of the predicted peptide-MHC score. **(A)** Workflow for *in silico* peptide MHC complex (pMHC) prediction based analyses. After selected SARS-CoV-2 proteins were cleaved *in silico* to produce peptide sequences, NetMHC-software was used to predict strong binding pMHC-combinations. Further processing included peptide filtering steps. **(B)** Numbers of 8 – 12 amino acid (AA) long peptides derived from the total viral proteome (ALL), highly expressed proteins (HIGHEX), the spike glycoprotein (SPIKE), and conserved sequences (CONS) are displayed. **(C)** Calculation of the predicted pMHC-score shown exemplarily for a given HLA genotype. Numbers of predicted strong binding pMHC complexes from the ALL pool for one exemplary *HLA* genotype are displayed. The sum of these numbers gives the locus-specific pMHC-score.

Next we tested the hypothesis that individuals whose HLA molecules can present more immunogenic viral peptides develop more efficient adaptive T-cell responses and thus experience less severe symptoms by the infection. For this purpose we predicted *in silico* binding affinities for peptide-MHC (pMHC) complexes derived from SARS-CoV-2 proteins for the given HLA repertoire of our study population. We defined four overlapping peptide pools derived from I) the entire viral proteome (ALL), II) highly expressed SARS-CoV-2 proteins (HIGHEX), III) the full-length spike glycoprotein (SPIKE), and IV) conserved regions (CONS) across different corona virus strains (**Figure 3**). Binding affinities were predicted for all allotypes represented in this patient cohort, resulting in 97,609,912 pMHC class I and 32,007,045 pMHC class II combinations. Strong binding pMHC complexes were defined by the NetMHC default rank score threshold of ≤0.5%.

**Figure 4:**
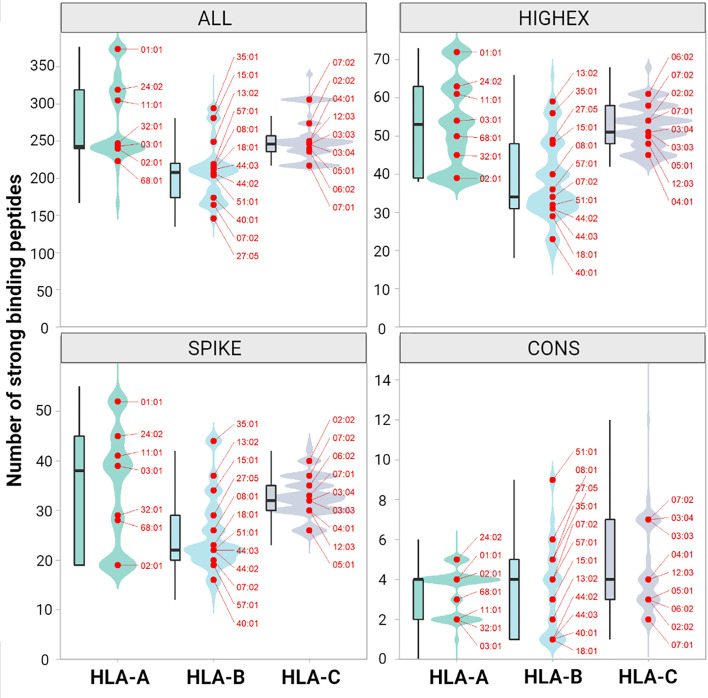
Numbers of predicted immunogenic peptides per HLA class I molecule. The figure shows the distribution of the number of binding peptides across all patients and both alleles for HLA-A, -B and -C. The distribution is shown both as kernel density plot and as boxplot for peptides derived from the entire peptidome (ALL, upper left panel), from highly expressed proteins (HIGHEX, upper right panel), from the spike glycoprotein (SPIKE, lower left panel) and from conserved regions (CONS, lower right panel). The most common alleles are highlighted as red dots. The ranks of the most common allotypes reflect their capacity to present immunogenic peptides of the respective peptide pool, whereas the relative amplitude reflects the frequencies of all allotypes with this specific capacity in the population. Changes in the ranks of the allotypes reflect a polarization of the respective peptide pools.

**Figure 5:**
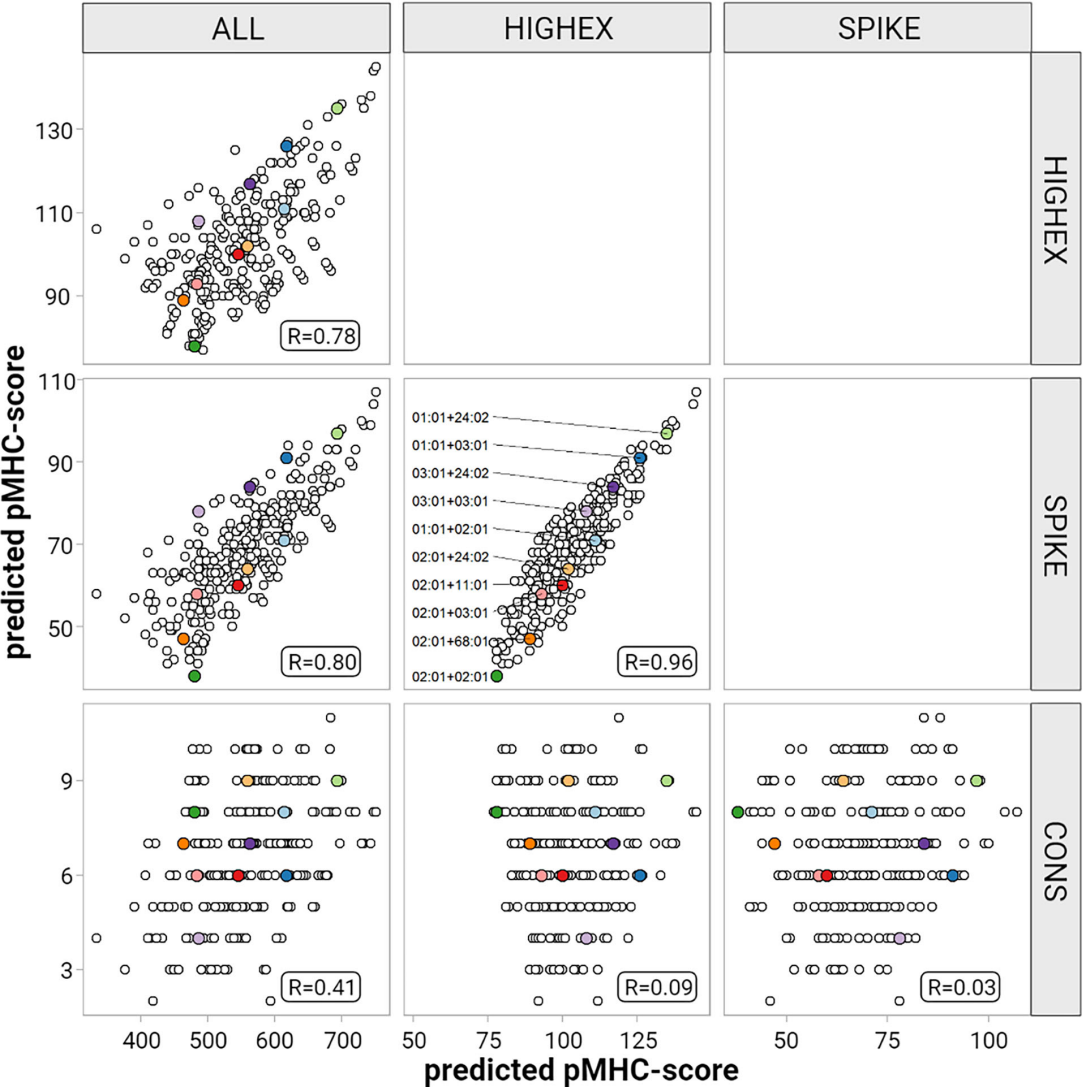
Correlation of the predicted pMHC-score for different peptide pools. This figure displays results for HLA-A pair-wise comparisons of the predicted pMHC-scores derived from the different peptide pools (entire peptidome (ALL), highly expressed proteins (HIGHEX), spike glycoprotein (SPIKE) and conserved regions (CONS)) for each patient. Pair-wise Pearson’s correlation coefficients are given in each panel. The ten most common HLA-A allele diplotypes are highlighted as colored dots.

For the ALL peptide pool the median numbers of predicted strong binding pMHC complexes per allotype were 243 for HLA-A (range, 167 to 377), 208 for HLA-B (range, 135 to 325), 246 for HLA-C (range, 217 to 340) summing up to 1,482 for HLA class I (range, 1,122 to 1,836), and 358 for HLA-DRB1 (range, 59 to 661) (see **Figure 4**). For the SPIKE peptide pool the corresponding numbers were 38 for HLA-A (range, 19 to 55), 22 for HLA-B (range, 12 to 45), 32 for HLA-C (range, 23 to 44) summing up to 184 for class I (range, 124 to 259), and 17 for HLA-DRB1 (range 0 to 99). The numbers of predicted strong binding pMHC complexes correlated tightly for the nested peptide pools ALL and SPIKE with correlation coefficients for HLA-A of 0.80 (p<0.001), for HLA-B of 0.79 (p<0.001), and for HLA-C of 0.55 (p<0.001), indicating that peptides derived from the 1,273-AA-long spike glycoprotein represent the entire virus proteome immunologically in a balanced way (see **Figure 5**). In contrast, *HLA* genotypes ranked differently with respect to numbers of predicted strong binding pMHC complexes derived from the much smaller CONS pool.

**Figure 6:**
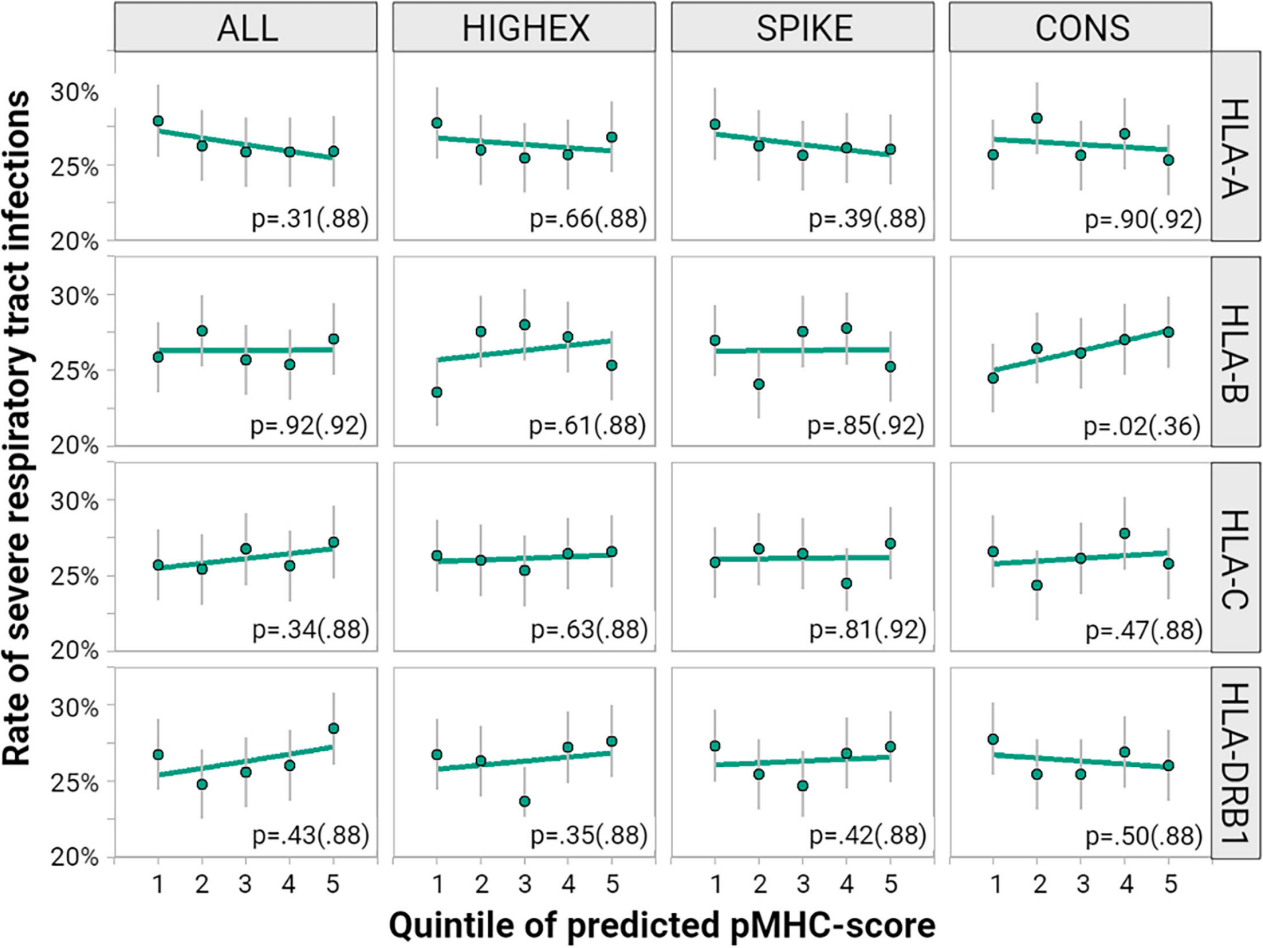
Impact of different peptide-MHC scores on the course of COVID-19. We correlated quintiles of predicted pMHC-scores calculated for HLA-A, -B, -C and -DRB1 for each of the peptide pools: the entire peptidome (ALL), highly expressed proteins (HIGHEX), the spike glycoprotein (SPIKE) and conserved regions (CONS). The figure displays the rate of severe respiratory tract symptoms (green dots) for each quintile along with 95% confidence intervals (gray lines). The green regression line represents a linear approximation of the rate of individuals with severe respiratory symptoms across the quintiles. The p values come from tests for a linear relationship based on multivariable logistic regression models with p values adjusted for multiple testing provided in brackets.

To correlate the individually predicted potential to present viral peptide with COVID-19 severity we calculated predicted pMHC sum scores for HLA-A, -B, -C, and -DRB1 genotypes for each individual of the study population. The rates of severe respiratory tract infections by five ranks of pMHC scores are displayed in **Figure 6**. We found no consistent correlation of the predicted pMHC scores with COVID-19 severity. Moreover, higher scores did not point homogenously toward less severe respiratory symptoms. Results for the exploratory phenotypes, symptomatic infections and respiratory hospitalizations were comparable (see **Supplementary Table S3**).

In a series of exploratory analyses, we re-evaluated the data by defining strong binding pMHC complexes by a NetMHC rank score of ≤2.0%. Furthermore, we tested various categorical scores and continuous scores with or without log transformation. Also, we tested the maximum number instead of the sum of pMHC scores in logistic regression models. No significant correlations were detected in these additional analyses (data not shown).

## Discussion

### Principal Findings

In a generally healthy working age population of 6,919 individuals who had recovered from SARS-CoV-2 infections we found no associations of specific *HLA* genotypes with the severity of the acute clinical course. Also, after correcting for multiple testing, we did not find significant associations of single allotypes with COVID-19 severity. These results suggest that individual *HLA* genotypes do not limit the immune response to SARS-CoV-2 infection as presumed by other studies (14–16, 36). Clinical factors such as age and body mass index had a far greater impact in our population compared to any analyzed factor describing the individual set of HLA molecules (34).

### Strengths and Weaknesses

To the best of our knowledge this study represents the largest cohort of patients with SARS-CoV-2 infections evaluated so far for a correlation of COVID-19 severity and *HLA* genotype. Using genetic information for *HLA-A*, *B*, *C*, and *DRB1* at a high resolution level, we tested a comprehensive set of biological concepts in a well defined, relatively homogenous cohort of 6,919 stem cell donors registered in Germany. Registered donors represent a relatively healthy subset of the general population aged between 18 and 61 years. Comorbidity would therefore hardly confound a potential *HLA*-genotype mediated effect on COVID-19 severity. A limitation of this study is the lack of information on the actual immune response in each single patient. We did not test directly for the serological or T-cell response to SARS-CoV-2 but used the severity of the clinical course as a surrogate for the quality of the immune response. Furthermore, *in silico* prediction of presumed immunogeneic pMHC complexes is limited by the fact that the multi-step process of peptide generation and presentation *via* HLA is not fully understood. Additionally, SARS-CoV-2 could interfere with the presentation of T cell epitopes (37). However, this part of the analysis relied only on the assumption that immunogenic T-cell epitopes are enriched among *in silico* predicted pMHC complexes. Under this condition, the predicted pMHC score is a suitable surrogate to investigate immunogenicity in relation to the *HLA* genotype.

This study is also limited by some other aspects. Unknown risk factors genetically linked to HLA genes had the potential to confound HLA effects. This limitation is inherent to all cohort studies. However, we can exclude large effects, described by odds ratios of 2 as chosen for the power calculations of our study. We considered effects of that size to be actionable because they had allowed predicting individual risks. Smaller effects (e.g. described by odds ratios of 1.3), which would still be of interest from an immunologic point of view, might be revealed with a sufficient power only when much larger cohorts will be analyzed. With respect to the clinical phenotypes, asymptomatic courses and the need for hospitalization clearly mark distinct COVID-19 severity. The identification of individuals with severe respiratory tract infections based on self-reported symptoms, however, is less stringent. Moreover, we were not able to test for genotype-phenotype correlations for specific clinical problems such as acute thromboembolic events, neurologic disorders or long-term complications.

Finally, lethal SARS-CoV-2 infections were not represented in this cohort by design. This number, however, is very small for a population with that age and sex distribution with a case fatality rate of approximately 0.11% according to epidemiological data.

### Comparison With Other Studies

Several studies which addressed T-cell responses in infected or convalescent patients suggested associations of specific *HLA* allotypes or genotypes with the severity of SARS-CoV-2 infections (14–16, 35). While our cohort of 6,919 individuals with SARS-CoV-2 infections outnumbered these studies, associations of single allotypes with clinical phenotypes withstanding stringent correction for multiple testing could not be revealed (**Table 2**). One reason might be that we applied systematic adjustment of p-values to keep control of the false discovery rate for different series of tests. Further, we attempted to reduce the number of statistical tests by evaluating biological concepts which allowed for the classification of *HLA* allotypes according to overarching principles. Also, we only tested for a supposed impact of specific *HLA* genotypes on COVID-19 severity and refrained from testing for an impact on the risk of contracting SARS-CoV-2. Unlike others, we excluded the possibility that contracting the infection could depend on the host *HLA* genotype while it clearly depends on exposition, variants, and the infective dose of SARS-CoV-2 (14, 38).

The diversity of *HLA-A, -B*, and *-DRB1* alleles can be reduced by grouping them into seventeen HLA supertypes according to functional or predicted structural similarities of their peptide-binding grooves. Certain HLA supertypes have been linked to more aggressive clinical courses of HIV infections, improved clearance of hepatitis C virus, and immune responses to hepatitis B vaccines (39–42). Although the size of this study was considerably larger than the studies on patients with HIV and hepatitis C, we did not find an impact of any HLA supertype on SARS-CoV-2 severity indicating that HLA supertypes do not uniformly predict outcome of viral infections (**Figure 1**). In contrast to SARS-CoV-2, HIV is also a chronic infection. HLA-restricted T-cell exhaustion, as shown for HIV, may thus not be relevant for SARS-CoV-2 (43). Moreover, HIV invades CD4^+^ T cells and impairs MHC class II-dependent immune responses, a feature not described for SARS-CoV-2. Therefore, immune responses and HLA associations may well differ between these infections.

Next, we investigated if the genetically restricted breadth of a T-cell response determines the severity of COVID-19. A heterozygote advantage in response to viral infections has been observed for humans infected with hepatitis B virus or HIV and for cynomolgus macaques infected with simian immunodeficiency virus (11, 12, 44). These effects are explained by a broader and more diverse immunopeptidome of individuals with heterozygous MHC genes compared to homozygous MHC genes. The concept of HLA evolutionary divergence measured with the Grantham Distance between two *HLA* alleles applies the same principle to heterozygosity (22). The Grantham Distance measures the divergence of the physicochemical properties of two *HLA* alleles and can be considered as a surrogate to test for the difference of the immunopeptidomes of these two alleles. Chowell et al. demonstrated in patients with malignant melanoma or non-small-cell lung cancer treated with immune checkpoint inhibitors which unleash T-cell immunity that patients with maximal heterozygosity at *HLA* class I loci and more divergent *HLA* class I genes showed improved survival (23, 34). Although the latter examples are taken from tumor immunology, they demonstrate the potential of the complementary concepts of heterozygote advantage and HLA divergence to predict T-cell immune responses. In contrast, our study showed neither a heterozygote advantage nor an impact of HLA class I divergence on COVID-19 severity (**Figure 2** and **Supplementary Table S2**).

Furthermore, we did not reveal significant correlations between individually predicted numbers of strong binding viral peptide MHC complexes and disease severity. Such correlations were postulated based on data from pure *in silico* studies which did not attempt to correlate individually predicted numbers of T-cell epitopes with clinical courses of SARS-CoV-2 infections (30). SARS-CoV-2 has one of the largest genomes (29.8 kb) among known RNA viruses - approximately 3 times the size of the genome of hepatitis C virus or HIV. Its open reading frames (ORF) encode numerous proteins which might serve as antigens for the adaptive immune response. We used NetMHCpan and NetMHCIIpan to generate SARS-CoV-2-derived peptide sequences and predicted their strength of binding to the individual set of HLA-A, -B, -C and -DRB1 molecules. A lower number of strongly binding peptide-MHC complexes did not correlate with severity of disease outcome in our data. Notably, the smallest sum of predicted strong binding peptide-MHC class I complexes for any given individual in our cohort was 1,122. Thus, simply the abundance of virus-derived T-cell epitopes may explain why neither single HLA allotypes, nor HLA supertypes or HLA homozygosity were not linked significantly to COVID-19 severity and guarantee that any individual set of HLA molecules allows for a robust T-cell response to SARS-CoV-2.

### Implications of This Study

The 1,273-AA-long spike glycoprotein may give rise to 6,208 peptides with a length of 8 to 12 AA. The median number of NetMHCpan predicted high-affinity peptide-MHC class I complexes derived from the spike glycoprotein for individuals in our cohort was 185 (range, 127 to 259). These numbers suggest that the spike glycoprotein itself is large enough to supply abundant T-cell epitopes for individuals with almost any given HLA genotype. The multitude of spike glycoprotein derived T-cell epitopes may also explain why effective immune responses to e.g. nucleoside-modified RNA vaccines which code for the full-length spike glycoprotein are not limited to few selected HLA genotypes but show efficacy rates of 95% (31, 32, 45). Moreover, our calculations suggest that few point mutations in the spike glycoprotein, as documented for the B.1.1.7 or the B.1.351 variant, will not result in substantial loss of immunogenic T-cell epitopes (46). Of greater concern are resulting changes of epitopes for antibodies which may lead to reduced neutralizing activity (47, 48).

### Unanswered Questions and Future Research

Scientifically, the pandemic represents a unique chance to catch the immune system in action responding to SARS-CoV-2. Only in the setting of a pandemic do patient numbers allow investigation of the genetics of the adaptive immune response. Our study is one of the largest HLA association studies carried out so far. Still, its size only allowed detecting strong associations between common allotypes and common phenotypes. Further collaboration in order to increase case numbers so that smaller but relevant effect sizes can be investigated is therefore urgently warranted. In addition, comprehensive genetic mapping of the immune proteasome and the peptide processing machinery as well as complete typing of *HLA* class II genes (including *DP* and *DQ*) would be highly attractive to gain deeper insights. Finally, genetic polymorphisms in immune response genes, including genes involved in innate immunity, might better explain the heterogeneity of COVID-19 disease courses.

In conclusion, this study provides unique insights into the potential of various *HLA* genotypes to mount efficient T-cell responses against SARS-CoV-2. Our data suggest that the proteome of SARS-CoV-2 is large enough so that abundant T-cell epitopes can be supplied for any *HLA* genotype. The individual *HLA* genotype is therefore no major factor which determines the course of the infection.

## Data Availability Statement

The original contributions presented in the study are included in the article/**Supplementary Material**, further inquiries can be directed to the corresponding author.

## Ethics Statement

The studies involving human participants were reviewed and approved by Institutional Review Board of the Technische Universität Dresden (IRB00001473). The patients/participants provided their written informed consent to participate in this study.

## Author Contributions

AS, SB, ST, FH, HB, and JSc designed the study. ST and FH performed systematic literature searches. ST, SB, RR, HB, JSc, and AS developed the health questionnaire. JSa, RB, JH, JM, and SB designed the database, validated data entry and export, and verified the data download. HB, SB, and JSa had access to the underlying data and verified their integrity. BF and CH performed the calculations of in-silico prediction. HB and JSc performed the statistical analyses. JSc, FH, and ST together wrote the first draft of the manuscript. All authors contributed to the article and approved the submitted version.

## Funding

DKMS initiated and conducted this study. The Federal Ministry of Education and Research (BMBF) supported the study by a research grant (COVID-19 call (202), reference number 01KI20177).

## Conflict of Interest

The authors declare that the research was conducted in the absence of any commercial or financial relationships that could be construed as a potential conflict of interest.

## Publisher’s Note

All claims expressed in this article are solely those of the authors and do not necessarily represent those of their affiliated organizations, or those of the publisher, the editors and the reviewers. Any product that may be evaluated in this article, or claim that may be made by its manufacturer, is not guaranteed or endorsed by the publisher.
