# Supplementary material for: Individual HLA-A, -B, -C, and -DRB1 Genotypes Are No Major Factors Which Determine COVID-19 Severity
Source: Front Immunol. 2021 Jul 26;12:698193. doi: 10.3389/fimmu.2021.698193 (PMC8350391; doi:10.3389/fimmu.2021.698193)

# SupplementAL MATERIAL

## Supplementary Table S2. Impact of HLA-A, -B, -C, and –DRB1 zygosity on SARS-CoV-2 infections

| **N of homozyg** | **N** | **(%)** | **Symptomatic infection** | | | | | **Severe respiratory symptoms** | | | | | **Respiratory hospitalization** | | | | |
| --- | --- | --- | --- | --- | --- | --- | --- | --- | --- | --- | --- | --- | --- | --- | --- | --- | --- |
|  |  |  | **Cases** | **OR** | **(95%-CI)** | **p** | **p_adj_** | **Cases** | **OR** | **(95%-CI)** | **p** | **p_adj_** | **Cases** | **OR** | **(95%-CI)** | **p** | **p_adj_** |
| **HLA-A** |  |  |  |  |  |  |  |  |  |  |  |  |  |  |  |  |  |
| 0 | 5,422 | (85.2) | 4,870 | 1 |  |  |  | 1,395 | 1 |  |  |  | 198 | 1 |  |  |  |
| 1 | 944 | (14.8) | 843 | 0.94 | (0.75-1.19) | .62 | .86 | 271 | 1.18 | (1.01-1.37) | .041 | .16 | 37 | 1.07 | (0.74-1.53) | .72 | .99 |
| **HLA-B** |  |  |  |  |  |  |  |  |  |  |  |  |  |  |  |  |  |
| 0 | 5,986 | (93.8) | 5,382 | 1 |  |  |  | 1,562 | 1 |  |  |  | 220 | 1 |  |  |  |
| 1 | 395 | (6.2) | 352 | 0.92 | (0.66-1.29) | .64 | .86 | 111 | 1.12 | (0.89-1.41) | .35 | .69 | 13 | 0.88 | (0.50-1.56) | .67 | .99 |
| **HLA-C** |  |  |  |  |  |  |  |  |  |  |  |  |  |  |  |  |  |
| 0 | 5,462 | (89.8) | 4,915 | 1 |  |  |  | 1,409 | 1 |  |  |  | 199 | 1 |  |  |  |
| 1 | 621 | (10.2) | 546 | 0.82 | (0.63-1.06) | .13 | .48 | 165 | 1.02 | (0.85-1.24) | .81 | .92 | 24 | 1.01 | (0.65-1.56) | .97 | .99 |
| **HLA-DRB1** | | |  |  |  |  |  |  |  |  |  |  |  |  |  |  |  |
| 0 | 6,125 | (92.1) | 5,509 | 1 |  |  |  | 1,606 | 1 |  |  |  | 228 | 1 |  |  |  |
| 1 | 524 | (7.9) | 466 | 0.88 | (0.66-1.18) | .39 | .78 | 140 | 0.99 | (0.81-1.22) | .94 | .94 | 21 | 1.00 | (0.63-1.58) | .99 | .99 |
| **HLA-A, -B, -C, -DRB1** | | |  |  |  |  |  |  |  |  |  |  |  |  |  |  |  |
| 0 | 4,134 | (72.3) | 3,724 | 1 |  |  |  | 1,065 | 1 |  |  |  | 149 | 1 |  |  |  |
| 1 | 1,171 | (20.5) | 1,039 | 0.86 | (0.70-1.07) | .18 | .36 | 289 | 0.95 | (0.81-1.10) | .48 | .51 | 39 | 0.91 | (0.63-1.31) | .62 | .82 |
| 2 | 251 | (4.4) | 218 | 0.74 | (0.50-1.10) | .14 | .36 | 70 | 1.10 | (0.82-1.47) | .51 | .51 | 13 | 1.36 | (0.75-2.45) | .31 | .82 |
| 3 | 115 | (2.0) | 104 | 0.99 | (0.52-1.88) | .97 | .97 | 44 | 1.74 | (1.18-2.56) | .005 | .02 | 6 | 1.39 | (0.59-3.26) | .45 | .82 |
| 4 | 46 | (0.8) | 41 | 0.89 | (0.34-2.31) | .81 | .97 | 9 | 0.71 | (0.34-1.49) | .36 | .51 | 0 | 0.00 | - | .97 | .97 |

HLA, Human Leukozyte Antigen; N, number; Homozyg, homozygous HLA loci; OR, odds ratio; CI, confidence interval; p, p-value; p_adj_, adjusted p-value; RTI, respiratory tract infection

## Supplementary Table S3. Impact of the predicted peptide-MHC scores on the course of SARS-CoV-2

| **Peptid pool** | **Score** | **N** | **Symptomatic infection** | | | | **Severe respiratory symptoms** | | | | **Respiratory hospitalization** | | | |
| --- | --- | --- | --- | --- | --- | --- | --- | --- | --- | --- | --- | --- | --- | --- |
|  |  |  | **OR** | **(95%-CI)** | **p** | **p_adj_** | **OR** | **(95%-CI)** | **p** | **p_adj_** | **OR** | **(95%-CI)** | **p** | **p_adj_** |
| **ALL** |  |  |  |  |  |  |  |  |  |  |  |  |  |  |
|  | A | 6,916 | 0.99 | (0.98-1.00) | .25 | .84 | 1.00 | (0.99-1.00) | .31 | .88 | 1.00 | (0.98-1.02) | .96 | .96 |
|  | B | 6,918 | 1.00 | (0.98-1.01) | .56 | .84 | 1.00 | (0.99-1.01) | .92 | .92 | 0.99 | (0.97-1.01) | .37 | .91 |
|  | C | 6,710 | 1.01 | (0.99-1.03) | .31 | .84 | 1.01 | (0.99-1.02) | .34 | .88 | 1.01 | (0.98-1.04) | .45 | .91 |
|  | A+B+C | 6,706 | 0.99 | (0.99-1.00) | .26 | .67 | 1.00 | (0.99-1.00) | .69 | .82 | 1.00 | (0.99-1.01) | .92 | .92 |
|  | DR | 6,857 | 1.00 | (1.00-1.00) | .45 | .84 | 1.00 | (1.00-1.00) | .43 | .88 | 1.00 | (1.00-1.01) | .56 | .91 |
| **HIGHEX** |  |  |  |  |  |  |  |  |  |  |  |  |  |  |
|  | A | 6,916 | 0.96 | (0.91-1.01) | .10 | .84 | 0.99 | (0.96-1.03) | .66 | .88 | 0.99 | (0.92-1.07) | .82 | .95 |
|  | B | 6,918 | 1.02 | (0.96-1.07) | .56 | .84 | 1.01 | (0.97-1.05) | .61 | .88 | 1.04 | (0.96-1.13) | .36 | .91 |
|  | C | 6,710 | 0.98 | (0.88-1.08) | .63 | .84 | 1.02 | (0.95-1.09) | .63 | .88 | 1.00 | (0.86-1.18) | .95 | .96 |
|  | A+B+C | 6,706 | 0.98 | (0.95-1.02) | .35 | .67 | 1.00 | (0.98-1.03) | .80 | .82 | 1.01 | (0.96-1.06) | .77 | .92 |
|  | DR | 6,857 | 1.01 | (0.99-1.02) | .32 | .84 | 1.00 | (1.00-1.01) | .35 | .88 | 1.00 | (0.98-1.02) | .78 | .95 |
| **SPIKE** |  |  |  |  |  |  |  |  |  |  |  |  |  |  |
|  | A | 6,916 | 0.96 | (0.92-1.01) | .11 | .84 | 0.99 | (0.95-1.02) | .39 | .88 | 0.99 | (0.92-1.07) | .83 | .95 |
|  | B | 6,918 | 1.01 | (0.94-1.09) | .71 | .84 | 1.00 | (0.96-1.06) | .85 | .92 | 1.03 | (0.92-1.16) | .57 | .91 |
|  | C | 6,710 | 0.99 | (0.86-1.14) | .87 | .90 | 1.01 | (0.92-1.11) | .81 | .92 | 1.10 | (0.89-1.37) | .38 | .91 |
|  | A+B+C | 6,706 | 0.98 | (0.94-1.02) | .29 | .67 | 0.99 | (0.97-1.02) | .66 | .82 | 1.01 | (0.95-1.07) | .81 | .92 |
|  | DR | 6,857 | 1.00 | (0.99-1.02) | .60 | .84 | 1.00 | (0.99-1.02) | .42 | .88 | 1.01 | (0.98-1.03) | .67 | .95 |
| **CONS** |  |  |  |  |  |  |  |  |  |  |  |  |  |  |
|  | A | 6,916 | 1.41 | (0.85-2.36) | .19 | .84 | 1.02 | (0.72-1.45) | .90 | .92 | 1.32 | (0.59-2.95) | .49 | .91 |
|  | B | 6,918 | 1.04 | (0.82-1.34) | .73 | .84 | 1.22 | (1.03-1.44) | .022 | .36 | 1.67 | (1.15-2.42) | .008 | .12 |
|  | C | 6,710 | 1.02 | (0.78-1.34) | .90 | .90 | 1.07 | (0.89-1.29) | .47 | .88 | 1.15 | (0.75-1.76) | .53 | .91 |
|  | A+B+C | 6,706 | 1.07 | (0.90-1.27) | .44 | .67 | 1.12 | (0.99-1.26) | .062 | .49 | 1.37 | (1.05-1.77) | .019 | .15 |
|  | DR | 6,857 | 1.02 | (0.94-1.09) | .69 | .84 | 0.98 | (0.93-1.03) | .50 | .88 | 0.87 | (0.77-0.99) | .031 | .24 |

HLA, Human Leukozyte Antigen; N, number; OR, odds ratio; CI, confidence interval; p, p-value; p adj, adjusted p-value; RTI, respiratory tract infection; ALL, entire peptidome; HIGHEX, highly expressed proteins; SPIKE, spike glycoprotein; CONS, conserved regions

The predicted pMHC score for each diplotype was analyzed as continuous variable in a multivariable logistic regression model for the risk of symptomatic infection, risk of severe respiratory tract infection (RTI) and risk of respiratory hospitalization. Effects were analyzed for different HLA class I and II molecules and the different peptide pools. The odds ratios represent the change in odds for every increase of 10 strongly binding peptides.

## Supplementary Figure S1. Flow chart.

The flow chart below displays exclusions of participants and the size of the final analysis sets. The questionnaire was successfully mailed to 4,440,895 registered DKMS donors and returned by 924,685 participants. 25 participants subsequently withdrew consent. Their data were deleted.

Additionally, data from 103 participants were excluded from data analysis for a variety of reasons: 64 participants reported a BMI of less than 10 kg/m² or more than 65 kg/m², 29 participants reported a test in December 2019, and 10 participants provided missing or inconsistent answers on testing status and disease symptoms.

Of the remaining 924,557 participants, 748,613 had never been tested for SARS-CoV-2. Participants who reported positive results from other tests (N=15,272), e.g. antibody tests, were excluded. In addition, 3,132 respondents did not know the results of their swab when they answered to the survey. The full analysis set thus contained 157,544 participants.

We defined two analysis populations:

The population to analyze the risk of contracting SARS-CoV-2 comprised 157,544 participants, 7,948 participants who had been tested positive and 149,596 individuals who had been tested negative.

The population to analyze risk factors for COVID-19 severity included all participants who had been tested positive, minus individuals who reported positive tests in August or September (N=1,029) because we could not assume that these participants had already recovered when they completed the health questionnaire and thus were not able to report maximum severity. This population comprised 6,919 participants.


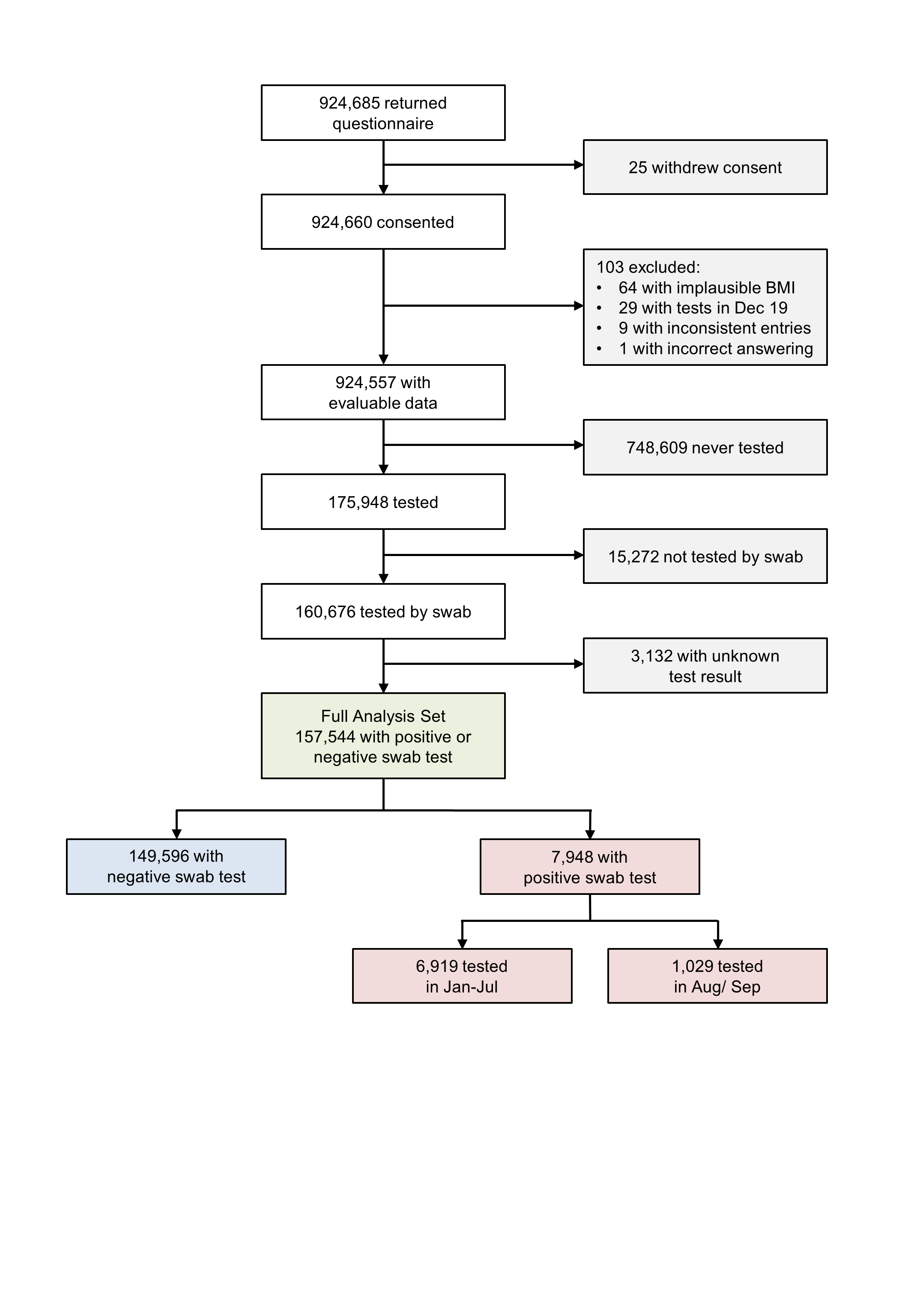


## Supplementary Figure S2. Distribution of Grantham distance

This figure displays the distribution of the HLA evolutionary divergences (HED) for all patients by using the Grantham distance (GD) metric of HLA-A, -B and -C allele pairs and as overall measure the mean GD of these three loci. The most common allele pairs are highlighted as red dots. Among the 10 most prevalent combinations of HLA-A, -B, and -C diplotypes in our cohort the highest GD values were obtained for HLA-A*03:01 and 24:02 (GD=10.7), HLA-B*07:02 and 44:02 (GD=14.3), and HLA-C*05:01 and 07:02 (GD=7.5).


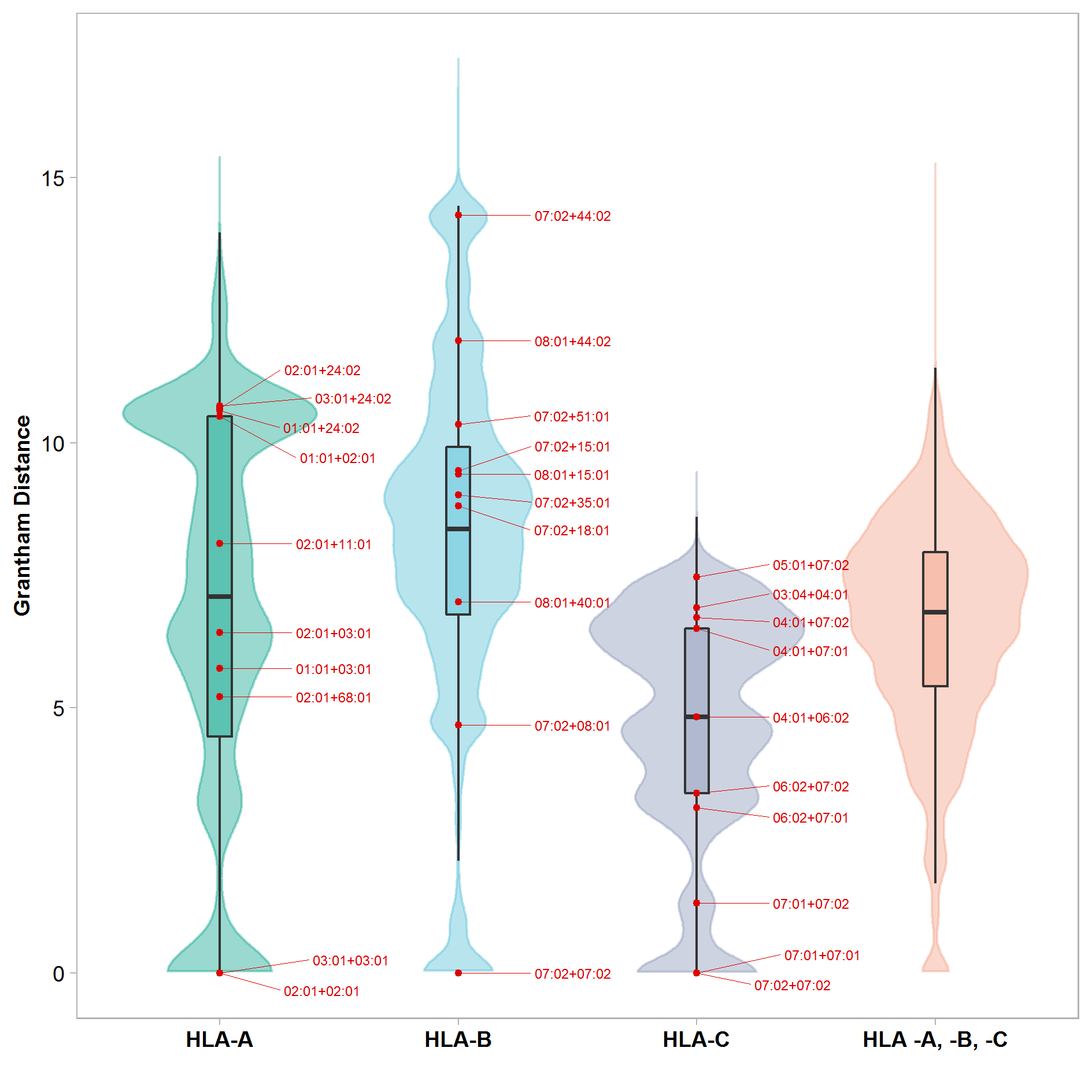

Supplement: Supplementary file 1 [file DataSheet_1.docx]
